# Data driven analysis of biomechanical factors associated with improved cross-country skiing performance

**DOI:** 10.3389/fspor.2025.1664279

**Published:** 2025-11-13

**Authors:** Alexander J. MacNeil, Tamar D. Kritzer, Alexis D. Napper, Damiano Fruet, Shawn M. Beaudette

**Affiliations:** 1 Department of Kinesiology, Faculty of Applied Health Sciences, Brock University, St. Catharines, ON, Canada; 2 Department of Kinesiology, Faculty of Sciences, McMaster University, Hamilton, ON, Canada; 3 Department of Industrial Engineering, University of Trento, Trento, Italy

**Keywords:** principal component analysis, support vector machine, joint power, ski propulsion techniques, motor coordination, wearable sensors, field testing, sport performance

## Abstract

The objective of this work was to implement a data-driven biomechanical approach that can assess the biomechanical determinants of cross-country skiing performance. To achieve this, full-body kinematic data were obtained and analyzed during over-ground cross-country skiing trials of varied efforts to quantify propulsion strategies, spatiotemporal coordination, drag, and joint power outputs. Eight athletes of varied skill levels were analyzed, encompassing a total of 5,568 movement cycles (i.e., propulsion strategies). To assess the many interacting modes of variation potentially associated with the skilled performance in cross-country skiing two complementary analyses were implemented. First, an automated objective classifier was trained on a subset of data to detect varied propulsion strategies associated with different athlete skill levels. Second, a principal component analysis was utilized to provide animated reconstructions of representative movement styles and relevant indicators of variance related to skill level. Results suggest that several factors were associated with skill-level including: (1) dominant propulsion strategy, (2) smaller frontal area, (3) reduced ski external rotation, (4) increased upper and lower body joint power. The data driven approaches implemented here can identify key features associated with cross-country skiing performance and have the capacity to be used in a sport-field setting to communicate efficient strategies to athletes.

## 1 Introduction

**Figure 1:**
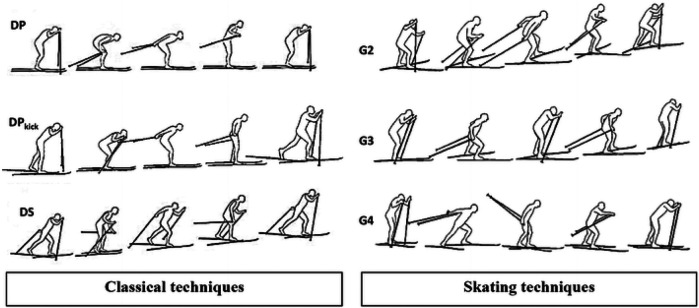
Depiction of cross-country skiing techniques from Herbert-Losier et al. (10) (CC BY 4.0). Left column represents classical technique and sub-techniques of double pole (top), kick double pole (middle), and diagonal stride (bottom). Right column represents skating technique and sub-techniques of G2 (top), G3 (middle) and G4 (bottom).

Cross-country skiing performance is reliant on the complex interaction of many body segments requiring the athlete to coordinate whole body movement and power to achieve enhanced propulsion speed and efficiency (1). The central nervous system must coordinate the activation and relaxation of upper and lower body musculature in a rhythmic nature to perform complex motor commands in a smooth, efficient manner [e.g., (2)]. Classical Skiing (CS) and Skate Skiing (SS) are the two main techniques utilized to navigate a cross-country skiing course, which have different sub-techniques (3, 4) (Figure 1). CS features sagittal plane movements in both the upper and lower body while the skis remain parallel. SS features a frontal plane push-off in the lower body with more abduction and external rotation at the hip, resulting in a posterolateral movement of the skis (3, 4). The two techniques are considered distinct disciplines, and athletes may exclusively train one style. SS is considered to be the faster technique and more difficult to learn (5). Each of these techniques have sub-techniques that can be used in different areas of a track if a skier needs to emphasize speed or force across varied terrain. On an uphill section, athletes may choose to use a sub-technique with a shorter cycle length and faster cycle rate (3). While on a flat or downhill surface, athletes may use a sub-technique with longer cycle lengths and lower cycle rates as they can glide after each push. One sub-technique in CS is the double pole technique, which is most used to generate speed with the greatest skiing economy (6). Selection and mastery of both CS and SS techniques may be an indicator of improved skiing performance (5).

Coaching cross-country skiing can be challenging and may take many years of practice to be able to detect errors and communicate them effectively to an athlete to elicit the proper changes in mechanics (7). Traditionally, coaches' provide feedback to athletes in the form of visual analysis, while leveraging verbal cues and prompts to alter mechanics. However, these cues may be subjective to a coaches own individual bias. Providing the athlete with objective visual feedback from a video or animation derived from an ensemble of their own movements may improve motor learning and skill acquisition (8). Since cross-country skiing is performed on a track that an athlete must navigate, this may restrict the ability for a coach to provide analyses across the entirety of a wide range of terrains since the coach may only be able to see the athlete's technique for a small portion of time. Motion capture technologies, such as those based on Inertial Measurement Units (IMUs), can capture full-body kinematic data over large distances, enabling coaches to monitor athlete technique even when out of visual range. This can permit coaches to collect data from athletes in sport-specific settings and around all areas of the track. These datasets can then be used to generate whole-body animations to visualize the technique of the athlete, provide quality feedback, and improve performance. Feedback can then be presented to a coach in a format that is interpretable (i.e., 3D human movement animation) rather than an artificially reduced format (i.e., discrete or time-varying metrics), which can discount whole-body multi-segment coordination strategies.

Fundamentally, cross-country skiing performance is defined by the ability of an individual to navigate a track in the shortest period of time. Cross-country skiing performance is affected by multiple potentially interacting and intersecting variables, thereby reducing the utility of analyzing specific sub-components (i.e., joints, or other specific movement features) in isolation (9, 10). Despite this, many previous studies have selected discrete parameters *a priori* for statistical analysis to infer performance (10–12). Given the dynamic nature of performance, there is a need to evaluate large volumes of time varying data in ecologically relevant (i.e., real-world, over-ground) scenarios. By leveraging tools rooted in data science, data structures can be examined to isolate biomechanical trends related to performance outcomes. One such tool capable of data reduction and feature identification is a principal component analysis (PCA). PCA is a statistical technique that reduces complex data sets into a series of orthogonal patterns of variances called principal components (PCs) (13). It helps to reduce and compare structures of variability within large time-varying datasets, to describe and discern objective waveform characteristics of high dimensionality between subjects or groups. PC scores describe the magnitude and timing variability of each data point obtained along a PC loading vector to understand the degree to which specific patterns of variance are observed (13). A main benefit of PCA in biomechanics is the capacity to use PC scores paired with subsequent multi-component reconstruction (MCR), to reconstruct data along specific modes of variation relating to an area of interest (i.e., performance, injury, etc.), providing the opportunity to develop full-body 3D animated tools for visual inspection. Reconstructing a full-body avatar may be an especially efficient way to provide athletes and coaches with an easy-to-interpret biomechanical analysis, communicating key technical components relating to high-dimensional data such as spatiotemporal kinematics. PCA and machine learning driven analyses have been previously used to examine these various factors associated with cross-country skiing performance (9, 14, 15), as well as across a variety of other human movements (2, 16, 17). PCA can assist researchers in examining a reduced feature set which lacks the redundancy or collinearity often associated with discrete biomechanical outcomes that are selected *a priori*.

Elite cross-country skiers can produce high skiing velocities with minimal kinematic variation to perform efficient propulsive movements (1). Identifying the variability of the individual may provide interpretable data relating to performance level and/or injury risk (18). For example, a decreased variability may be indicative of enhanced performance but can also lead to a risk of chronic injury as the same bodily tissues are repeatedly used to perform a given task. An increased task variability can relate to poorer performance and can lead to a risk of acute injury (18). The ability of an athlete to self-regulate their kinematic variability while settling on an optimized strategy given environmental constraints may be an important factor in reducing injury risk while maximizing performance.

The overall objective of this work was to (1) develop an objective method to identify and classify discrete cross-country skiing technique types (2) implement a data-driven biomechanical approach that can objectively assess the kinematic and kinetic cross-country skiing characteristics associated with performance through the analysis of a large dataset. Given the data-driven, exploratory nature of this research, it can be considered hypothesis generating.

## 2 Materials and methods

### 2.1 Participants

Eight participants of varied skill levels completed an over-ground skiing protocol consisting of three trials of varied effort: easy, medium, hard. On the field, participants self-imposed the effort to correspond to the targeted heart rate (HR) zones. Easy effort corresponded to zone 1 (50%–60% max HR), medium effort to zone 3 (60%–80% max HR), and hard effort to zones 4/5 (80%–100% max HR). Demographic variables for all participants are provided in Table 1. The recruited subjects were healthy adult volunteers with no declared cardiovascular, respiratory, or autonomic control diseases. They were required to complete a cross-country skiing outing of at least 10 km. Detailed information regarding the inclusion criteria was provided to all participants, and the trial commenced only after each participant had provided a signed informed consent form. All participants who did not meet the inclusion criteria were excluded from the study. The protocol for the current study was reviewed and approved by the Research Ethics Committee of the University of Trento (protocol number 2023-021) in accordance with the Declaration of Helsinki. All participants reviewed and signed informed consent prior to data acquisition. Athlete skill level was self-reported by participants based on the following criteria:

- Beginner: individuals who engage in recreational cross-country skiing approximately once per week during the winter season without structured training programs.
- Intermediate: amateur athletes who train at least twice weekly during the winter season, incorporating structured training sessions, and regularly participate in organized local or regional cross-country competitions.
- Advanced: highly skilled amateur athletes who train consistently throughout the year, with a focus on performance improvement. They participate in regional or national level competitions.
- Elite: professional athletes from the Fiamme Gialle Sports Group of Guardia di Finanza, who train year-round with a dedicated coaching staff and compete at national and international levels.

**Table 1:** Participant demographic information, including the overall elapsed time and number of *cycles* extracted from each trial across all participants.

ID	Sex	Age (years)	Height (cm)	Skill level	Trial [time (s), Num cycles]		
					Easy	Medium	Hard
P3	M	38	175	Beginner	253.5 s *226 cycles*	230.8 s *224 cycles*	237.5 s *234 cycles*
P12	M	33	183		248 s *510 cycles*	218 s *443 cycles*	208.7 s *360 cycles*
P13	M	13	165	Intermediate	155.5 s *180 cycles*	131.3 s *179 cycles*	113.7 s *156 cycles*
P14	M	13	151		165 s *192 cycles*	129.4 s *164 cycles*	123.6 s *173 cycles*
P7	F	30	172	Advanced	240.5 s *318 cycles*	154.9 s *246 cycles*	193.5 s *270 cycles*
P8	F	27	171		155.5 s *257 cycles*	132 s *236 cycles*	124.5 s *207 cycles*
P5	F	23	164	Elite	184 s *250 cycles*	114 s *200 cycles*	109.4 s *183 cycles*
P6	M	21	200		138.6 s *143 cycles*	114.3 s *107 cycles*	100 s *119 cycles*

### 2.2 Materials

Participants wore an XSens Link sensor suit consisting of 17 inertial measurement unit (IMU) sensors on the body and four additional sensors located on the ski poles (×2) and skis (×2) that sampled kinematic data at 240 Hz (Movella Inc., Henderson, NV). The XSens Link was available in six sizes, and each participant wore the suit that best fit their specific body characteristics. Sensors and cables were affixed to the body using dedicated housings placed on the suit. The sensor on the foot was directly attached with a strap around the ski boot, positioned on the instep. IMUs on the poles were fixed immediately below the pole handles. IMUs on the skis were placed in front of the ski bindings. Once all sensors were placed, the calibration process was performed using MVN software. The guided procedure first required the measurement of specific sensor positions (e.g., the height of the IMU on the poles), followed by the repetition of specific movements. This allowed for the exact alignment of each sensor's orientation with its corresponding body segment. The experimenter assisted with the donning procedure and the placement of sensors on the poles and skis, ensuring correct sensor positioning and connection to the main acquisition unit. Each participant was allowed to use their own equipment, consisting of skis, ski boots, and poles, avoiding the time required for familiarization with equipment and binding settings, thus preventing the introduction of any other familiarization-related variables into the study. Therefore, sensors were positioned individually for each subject for each acquisition. Elite skiers used skating skis to complete the course (due to training of that technique later in the day), the remaining equipment types were not recorded. Participants also wore an MTi 680-G GNSS (Movella Inc., Henderson, NV) unit that collected global positioning data at 4 Hz, which was used to reconstruct track topography. All data were logged into a portable data pack, ensuring synchronization, and subsequently downloaded using XSens MVN Analyze software (version 2022.0).

### 2.3 Experimental protocol

All data were acquired along a standardized track located at the Cross-Country Stadium of Lago di Tesero (Trento, Italy). The track used for data collection was an oval shape, primarily flat, with one short climb and one short descent on each long side (∼20 m elevation change). Data were captured over two days with an average temperature of −7.3°C ± 3.8°C, an average wind speed of 9.3 km/h ± 2.7 km/h and an average snow depth of 68.8 cm ± 2 cm. Following an initial warm-up loop for familiarization with the environment and acquisition equipment, participants completed three separate rounds of the track at increasing effort levels (1–3× easy, 2–3× medium, 3–3× hard) with the hard being a maximum effort trial. Trials were completed in order of increasing power, ensuring all athletes were adequately warmed up before the maximum effort (i.e., final) laps. Within each course loop, participants were required to employ a range of sub-techniques based on their preferred main technique (Figure 1): G2, G3, G4 for skating; double pole, kick double pole (flat), double pole, kick double pole, diagonal stride, or herringbone (diagonal stride with skis angled outward and without a glide phase for uphill) for Classic; free glide or standing glide (i.e., skis parallel during downhills and without any arm or leg push-off) for both Skating and Classic. All downhill sections were completed in a tucked position (free glide).

### 2.4 3d track reconstruction

**Figure 2:**
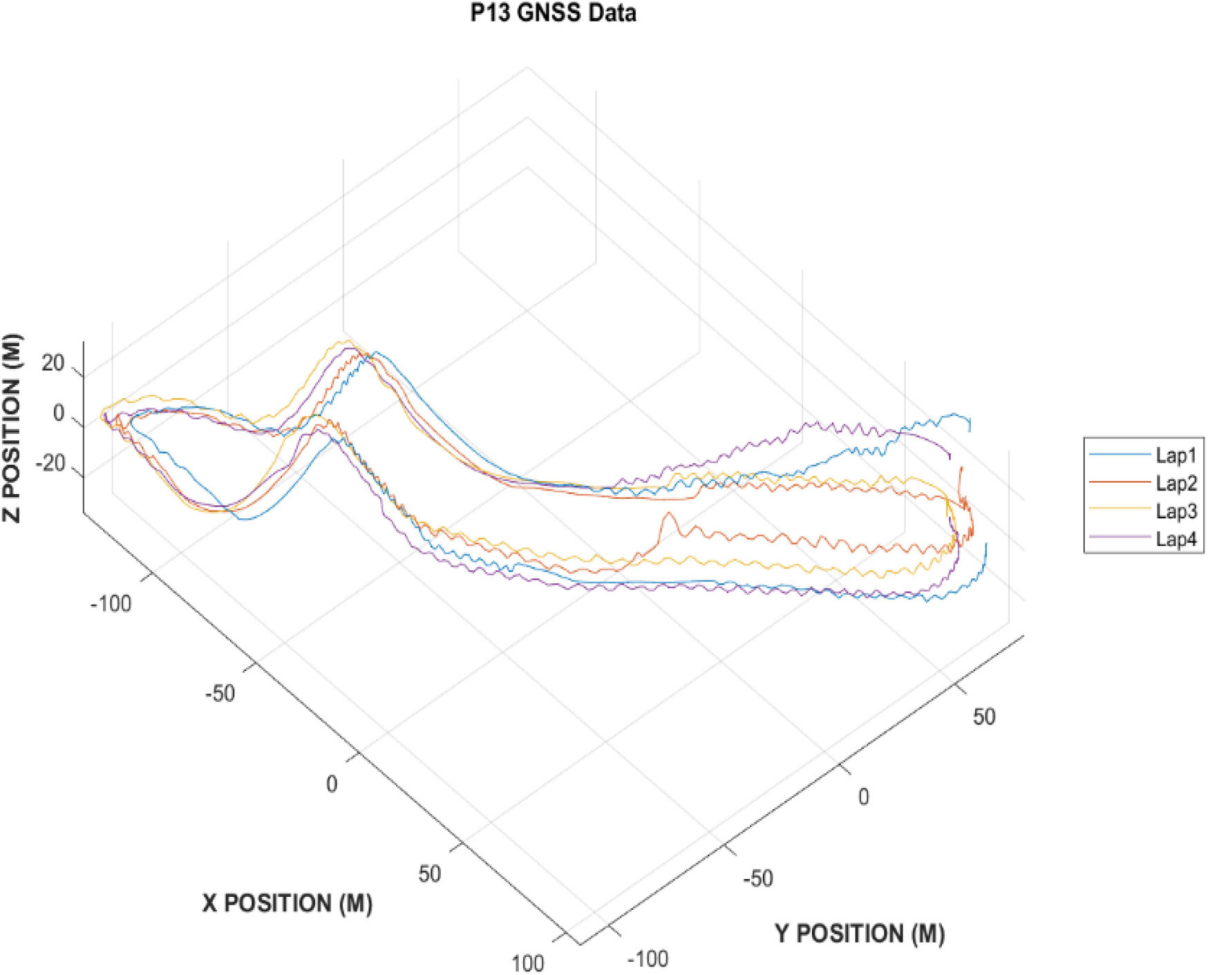
3D track reconstruction from P13 GNSS data.

To reconstruct a 3D representation of the cross-country skiing track, the GNSS data from one participant (P13) were used. Specifically, the local x and y coordinates were used in addition to the elevation relative to sea level. Following unit conversion, track revolutions were segmented and demeaned to remove any spatiotemporal drift within the GNSS data. A visual representation of the 3D track topography is depicted in Figure 2.

### 2.5 Pre-processing & joint power analysis

Raw IMU sensor data were high-definition (HD) reprocessed using MVN Analyze (version 2022.0), with all processed kinematic data being exported as .C3D files for further analysis elsewhere. To facilitate the analysis of joint power, whole-body kinematic data of all participants were imported into Visual 3D (C-Motion, Germantown MD, USA). For all participants and trials, a time-varying scalar metric of joint power (summed across all component axes of each joint) was extracted for the shoulders (bilaterally), thorax and abdomen (i.e., RTA), and knees (bilaterally). All joint power data were then normalized to participant mass and exported to MATLAB (The MathWorks, 2022a) for additional analysis.

**Figure 3:**
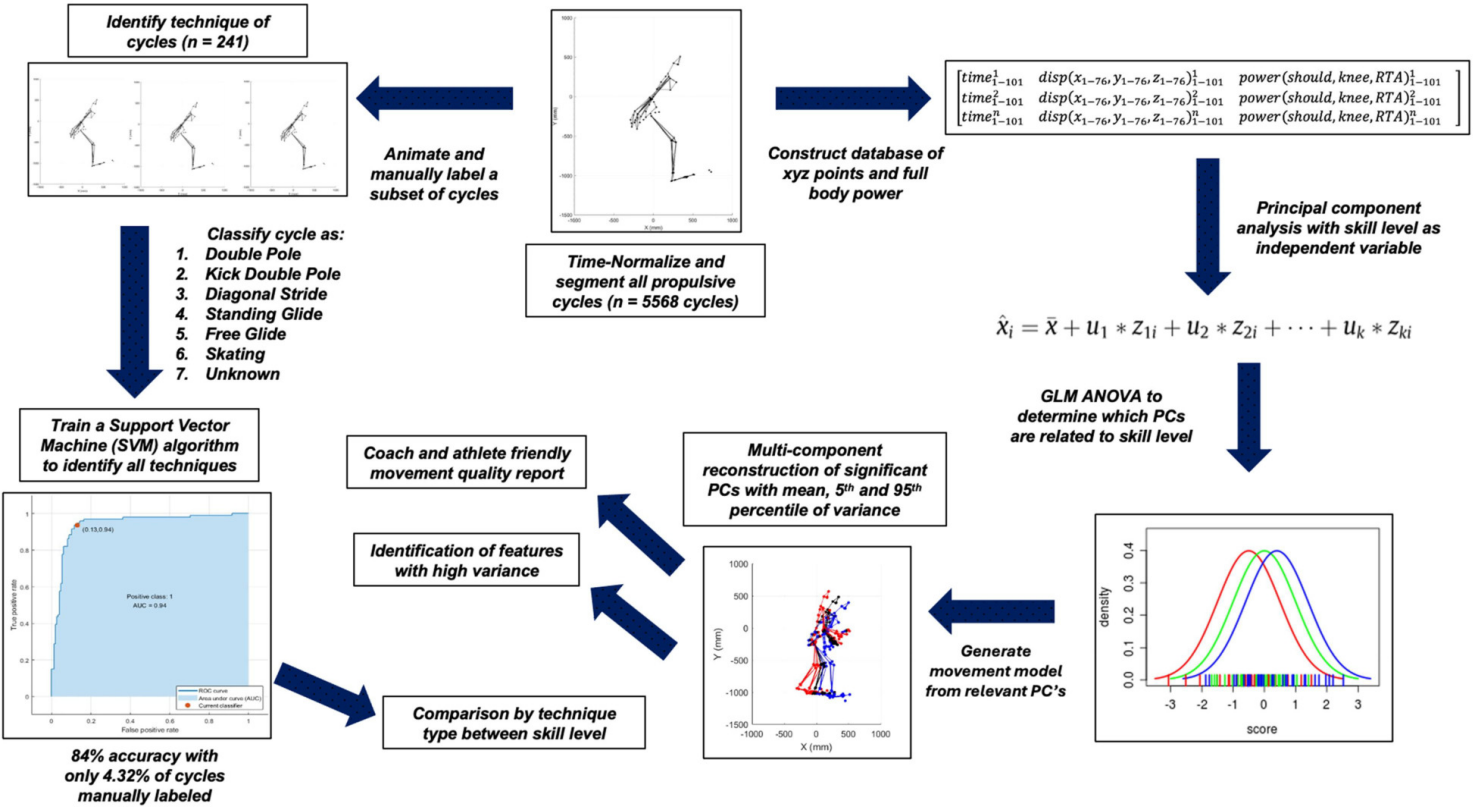
Flowchart depiction of the data analysis process.

Coordinate (C3D) data and joint power outputs were processed and analyzed using a custom MATLAB script. First, point cloud data consisting of all 76 (x, y, z) data points were aligned to a 3D local reference frame located to each participants' pelvis, at each instant in time. Specifically, a 3 × 3 rotation transformation matrix was generated at every frame to represent the 3D misalignment between the participant's pelvis local coordinates and global coordinate system, which was then subtracted, such that all data were represented with respect to the local reference frame of the pelvis. This was to ensure that each participant would be facing the same direction for all movements, even when changes in heading occurred. Next, 3D locations of all data points were referenced to the location of the T12 spinous process to remove effects of small linear translations of the point cloud throughout the trial (Figure 3).

**Figure 4:**
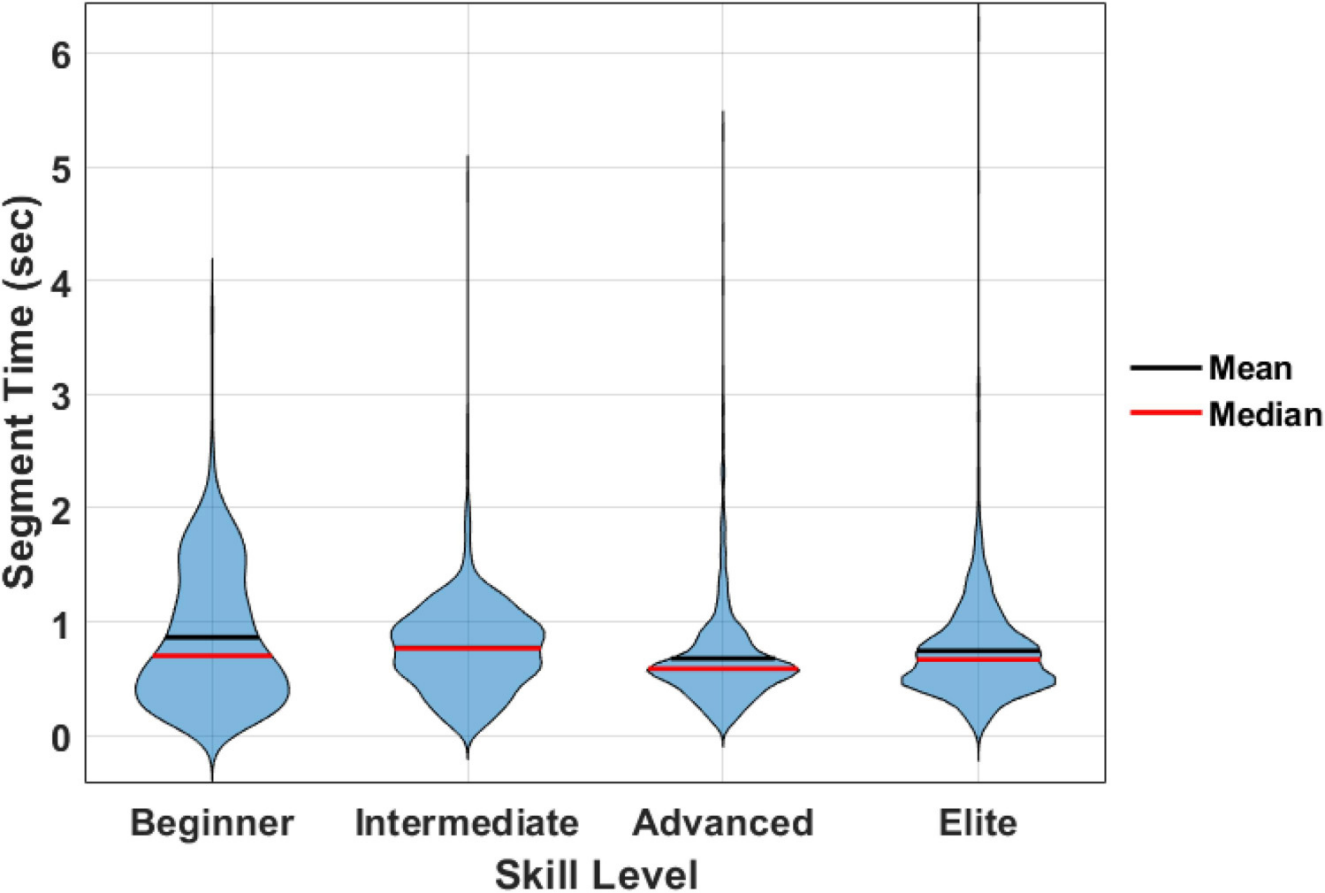
Cycle cadence characteristics across skill level.

Individual movement cycles (i.e., propulsion strategies) were automatically segmented by referencing the displacement of the left toe marker derived from the XSens MVN kinematic body model. Specifically, all Euclidean Norm displacement peaks exceeding 1 cm were used to partition individual propulsive movements. These criteria were selected following visual analysis of the 3D kinematic data of several participants/trials to identify a parameter capable of segmenting a wide range of propulsion strategies (i.e., double-pole, kick double-pole, etc.). Following cycle segmentation, all 3D coordinate data and joint-power time series data for each cycle were time-normalized to 101 data points by evenly resampling data from a Piecewise Cubic Hermite Interpolating Polynomial (PCHIP). Next, mean cycle durations were interpreted across athlete skill levels to ascertain any differences in athlete cadence across skill levels (Figure 4). Point cloud data within each cycle were retained for further analysis and the development of an automated propulsion strategy classification framework.

### 2.6 Principal component analysis (PCA)

Once aligned, demeaned, segmented, and time-normalized a structured database was developed of the following format:

$$\left[\begin{array}{ccc} {\mathrm{time}}_{1\text{-}101}^{1} & \mathrm{disp}(x_{1\text{-}76},y_{1\text{-}76},z_{1\text{-}76}{)}_{1\text{-}101}^{1} & \mathrm{power}(\mathrm{shoulder},\mathrm{knee},\mathrm{RTA}{)}_{1\text{-}101}^{1}\\ {\mathrm{time}}_{1\text{-}101}^{2} & \mathrm{disp}(x_{1\text{-}76},y_{1\text{-}76},z_{1\text{-}76}{)}_{1\text{-}101}^{2} & \mathrm{power}(\mathrm{shoulder},\mathrm{knee},\mathrm{RTA}{)}_{1\text{-}101}^{2}\\ {\mathrm{time}}_{1\text{-}101}^{n} & \mathrm{disp}(x_{1\text{-}76},y_{1\text{-}76},z_{1\text{-}76}{)}_{1\text{-}101}^{n} & \mathrm{power}(\mathrm{shoulder},\mathrm{knee},\mathrm{RTA}{)}_{1\text{-}101}^{3} \end{array}\right]$$

All rows correspond to individual cycles (from all participants), and all columns correspond to time-normalized, time, x/y/z coordinate, and scalar power time-series data (summed bilaterally for upper and lower body). All subsegments of the data representing different units of measurement (i.e., millimeters, seconds, or W/kg) were feature (i.e., amplitude) normalized by multiplying by scalar values such that the range of amplitude for each time-series metric occupied approximately the same range of values. A principal component analysis (PCA) was conducted on the database to deconstruct the variance into principal components (PCs). As a means of further data reduction, only PC scores explaining a cumulative 90% of variance within the data were retained for further statistical analysis (17 PCs, Table 2). PCA preserves the variability of multivariate datasets while reducing data dimensionality (19). Many studies use features extracted by PCA as input to machine learning models [e.g., (2, 16, 17, 19)]. In this study, we used PCA to extract data features and to eliminate redundant variables. Following PCA, propulsion cycle waveforms were reconstructed to visualize the biomechanical meaning of all PC scores associated with improved skill level.

**Table 2:** Statistical analysis depicting PC scores related to skill level.

PC number	Explained variance (%)	Cumulative explained variance (%)	F statistic	*p*-value
1	23.4	23.4	249.08	<.0001
2	20.5	43.9	331.61	<.0001
3	10.7	54.5	3,919.97	<.0001
4	6.3	60.9	372.32	<.0001
5	4.8	65.6	9.59	0.0020
6	3.8	69.4	13.30	0.0003
7	3.4	72.8	102.16	<.0001
8	2.8	75.6	1.19	0.2761
9	2.7	78.3	1.55	0.2126
10	2.4	80.7	788.02	<.0001
11	2.2	82.9	101.16	<.0001
12	1.7	84.6	111.57	<.0001
13	1.5	86.2	1,421.41	<.0001
14	1.3	87.5	692.78	<.0001
15	1.1	88.5	440.51	<.0001
16	1.0	89.5	4.49	0.0341
17	0.9	90.4	251.06	<.0001

Highlighted row indicates statistically significant stepwise relationship with skill level.

### 2.7 Automated propulsion strategy classification

A subset of individual propulsive cycles was animated and manually labelled as either: (1) double pole, (2) kick double pole, (3) diagonal stride, (4) standing glide, (5) free glide, (6) skating, or (7) unknown. Specifically, 241 cycles were manually labelled from the database of 5,568 cycles (4.32% of available data). Given the comparatively low number of skating cycles, all these sub-techniques (i.e., G2, G3, G4) were grouped together. The cycles were selected in an effort to manually label a sufficient number of examples of each strategy, without the need to manually label all cycles. Following this, the labels and PC scores (1–17) were used to train a quadratic support vector machine (SVM) automated classifier. Once trained, this classifier was used to automate the classification of propulsion strategies used across all 5,568 cycles.

### 2.8 Statistical analysis & training tool 3d reconstruction

A general linear model (GLM) one-way ANOVA was implemented with athlete skill level (i.e., beginner to elite) as the independent variable, and PC scores (1–17) as the dependent variables. Further, violin plots were generated to depict the relationship between PC scores and athlete skill levels. Those identified as having systematic (i.e., stepwise), and significant (i.e., *p* < 0.05) relationships with skill-level were used as inputs into a multi-component reconstruction framework with representative PCs reconstructed as either the 95th or 5th percentiles for PCs displaying positive or negative relationships with skill-level (2):

$${\hat{x}}_{\mathrm{Beginner}/\mathrm{Elite}}=\bar{x}+u_{1}\;*\;z_{95/5}+u_{2}\;*\;z_{95/5}+u_{3}\;*\;z_{95/5}+\ldots \;u_{n}\;*\;z_{95/5}$$

Where $\hat{x}$ represents the reconstructed data, $\bar{x}$ represents the mean, *u* represents the PC loading vector and *z* represents the 95th or 5th percentile PC score. This framework animates the primary modes of variation present within the dataset which are systematically (and significantly) related to athlete skill level.

## 3 Results

The distribution of skill levels, and segmented propulsion cycles from each athlete are presented in Table 1. Violin plots of cadence characteristics across skill levels revealed changes in the distribution of cycle times across skill levels (Figure 4). Although differences in cycle time distributions existed, there was no significant difference in mean cycle time across the skill levels.

### 3.1 Assessment of technique and SVM classification

**Figure 5:**
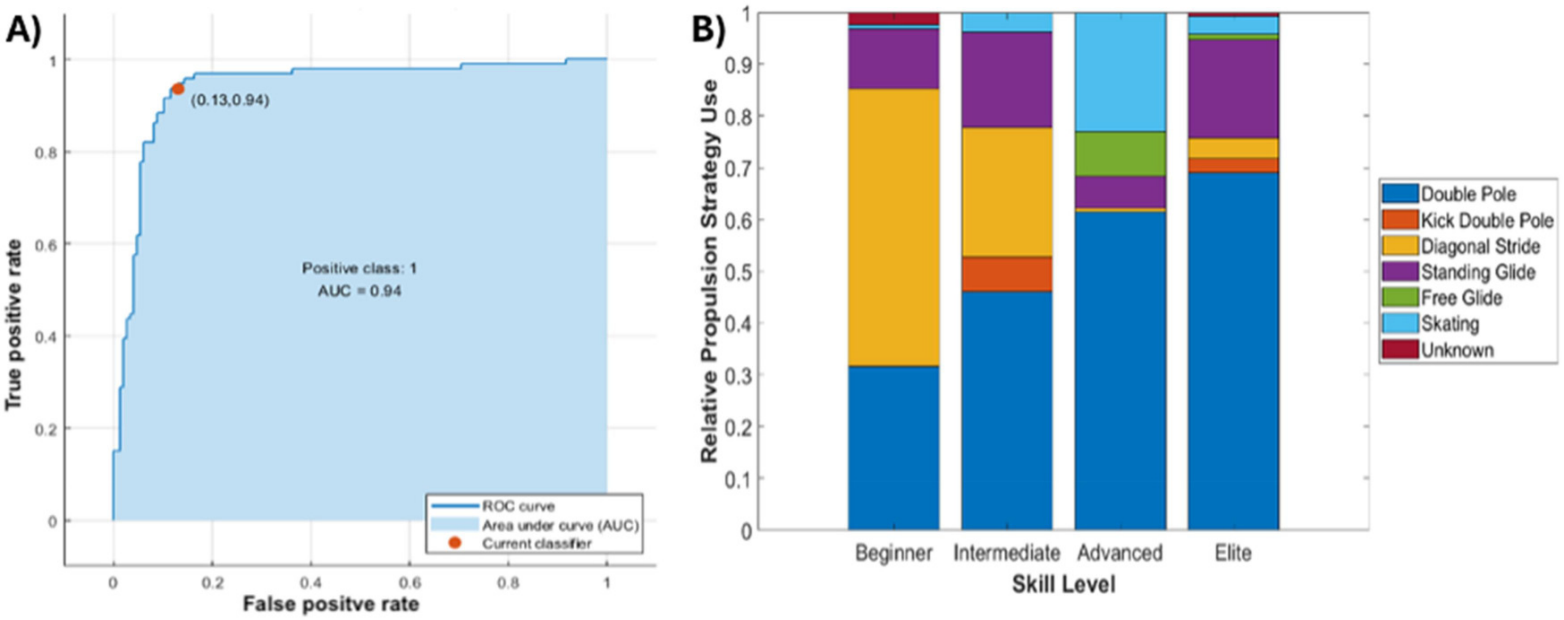
**(A)** ROC curve for propulsion strategy prediction model. **(B)** Depiction of propulsion strategies across skill levels.

The SVM classifier trained to identify the propulsion cycle techniques demonstrated 84% accuracy, with an AUC test statistic of 0.94 (Figure 5A). Following automated classification, the 5,568 cycles were plotted to depict the relative use of each propulsion strategy across skill levels (Figure 5B). This analysis suggests that the beginner skiers relied more on the diagonal stride technique than advanced and elite skiers, who relied more on a skating technique. Further, as the skill level increased, so did the frequency of usage of the double pole technique.

### 3.2 Assessment of kinematics and characteristics related to skill level

**Figure 6:**
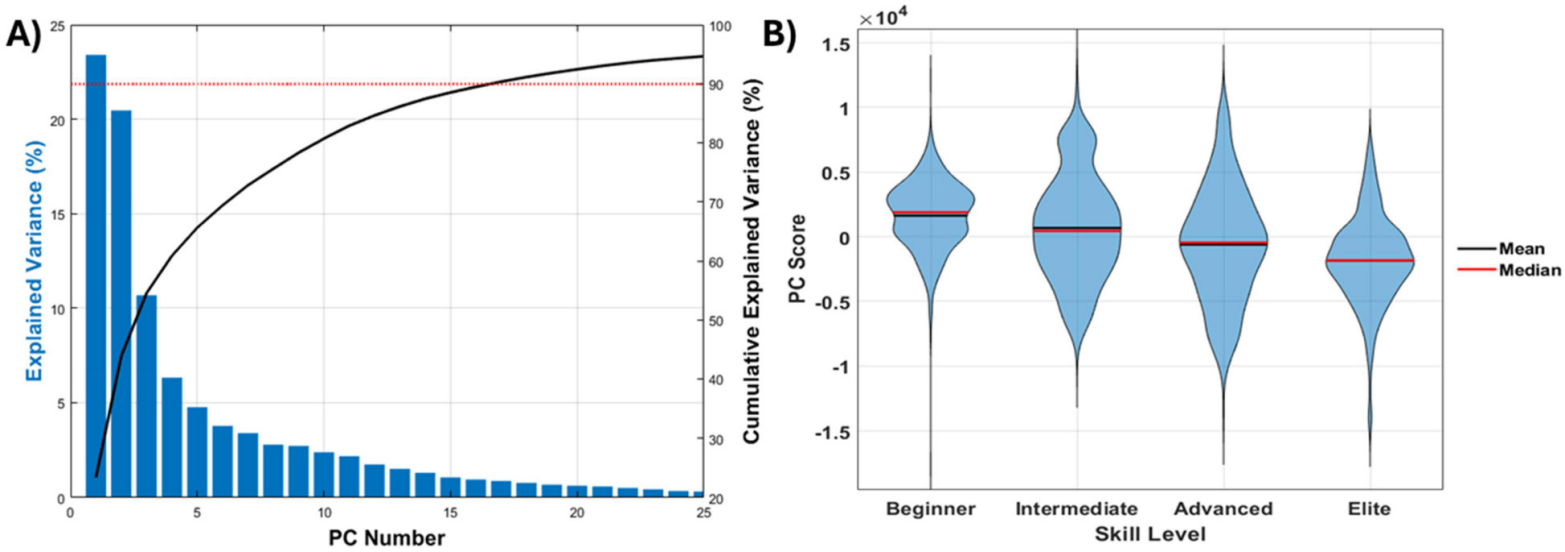
**(A)** Scree plot depicting explained variance statistics from PCA. **(B)** Statistically significant effect of skill level on PC10.

The assessment of the kinematic data included the first 17 PCs which explained a cumulative variance of 90.4% in the pre-processed dataset (Figure 6). A GLM model one-way ANOVA assessing the effect of skill level on the PCs revealed that PC4 (*p* < .0001), PC6 (*p* = .0003), PC10 (*p* < .0001), PC11 (*p* < .0001), PC15 (*p* < .0001) were significantly and systematically associated with skill level (Table 2).

**Figure 7:**
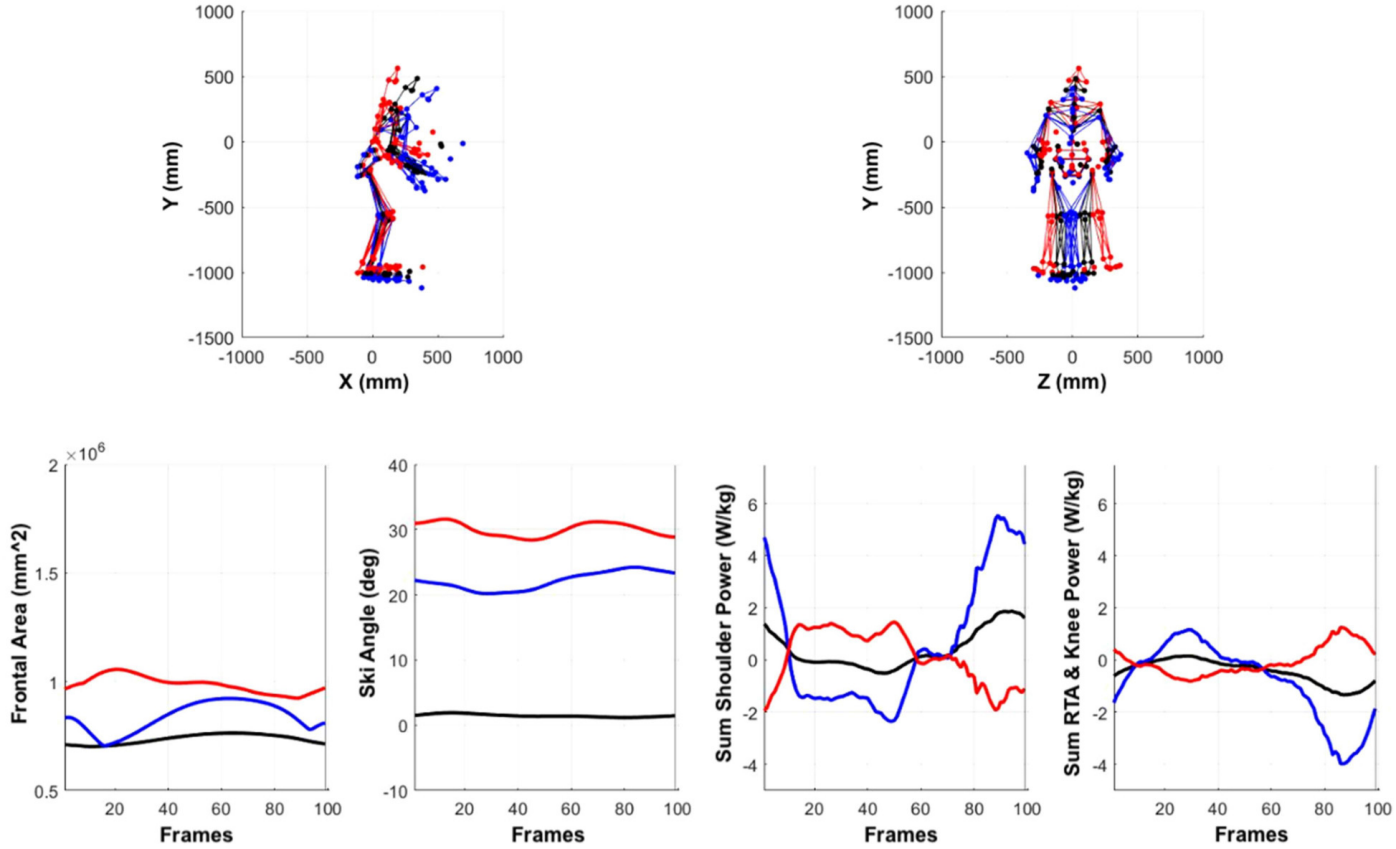
Multi-component reconstruction (PC 4, 6, 10, 11, and 15) depicting whole-body spatiotemporal differences associated with skill level. Red and blue figures represent extremes in variance associated with lower (red) and higher (blue) skill levels. Ski angle was calculated from transverse plane rotation of ski sensor data. Frontal area was estimated as a sum of two rectangular frontal planes derived from the maximum mediolateral and anteroposterior point cloud data (representing the upper and lower body respectively). RTA and knee power are summed together to represent gross lower body power.

Multi-component reconstruction depicting whole-body spatiotemporal differences associated with skill level was completed using PCs 4, 6, 10, 11, and 15. Collectively, these PCs represent 15.8% of the total variance in the dataset (Figure 7). Functional interpretation of kinematic PCs through MCR provided over-emphasized visualization of the spatiotemporal modes of variation between “Elite” and “Beginner” groups for means of visualization. Reconstructed time-series from these reconstructed data include outcomes related to frontal area, ski angle, and upper/lower body power. Results of the multi-component reconstruction indicate that elite athletes generate a greater upper body peak power (bilaterally) than beginner athletes. In comparison, elite athletes also tend to occupy less frontal area and have a reduced ski angle, resulting in a reduced drag.

## 4 Discussion

The overall objectives of this work were to (1) develop an objective method to identify and classify discrete cross-country skiing technique types (2) implement a data-driven biomechanical approach that can objectively assess the kinematic and kinetic cross-country skiing characteristics associated with performance through the analysis of a large dataset. Currently, coaches use subjective observations of technique through a skilled, trained eye to develop athletes to the elite level (7). The model developed in this study demonstrates that implementing data-driven biomechanical approaches, such as a PCA and SVM, can assist in the objective identification of phenotypic differences between athletes of differing skill levels. One of the strengths of using these approaches is the capacity to represent meaning and context of large volumes of time-varying data obtained from real-world scenarios. As such, the analysis implemented here used every movement from every trial and every athlete to inform the current results and demonstrate powerful data reduction capabilities. This however may not have accounted for differences that are a result of comparing mechanics across technique types. A practical application of this model would be to first use the SVM classifier to identify all of the cycles by a specific technique type, then perform a statistical analysis on the mechanical features obtained from this stratified dataset. This is, of course, an interesting area for further study. The findings regarding cycle cadence not being an indicator of better performance (Figure 5) align with most studies on cross-country skiing performance and faster cycle cadence may lead to worse skiing economy (1). This demonstrated that a skiing performance analysis would need to capture spatiotemporal variations to identify differences across skill level.

The results from the SVM automated classification of sub-techniques suggested that the double pole sub-technique was one of the favored strategies for producing the greatest speed and the fastest time to complete the track associated with higher skill levels. The diagonal stride sub-technique was associated with less experienced skiers and therefore slower speeds. Complementing these findings were the results of the MCR, where differences between skill level may be better explained by upper and lower body power outputs during propulsion. As each of the upper and lower body power metrics are informed by the sum of a different number of joints, the results suggest larger power contributions from the shoulder and upper body components in all skiers (especially those classified as elite). This aligns well with the type of sub-techniques that were selected by different skill levels. It has previously been demonstrated that there are greater contributions to speed and power from the upper body joints (shoulder and waist) than the lower body joints (12, 18). This may suggest that the upper body is responsible for generating propulsion while lower body joints create stability and influence direction. This is unsurprising given the recent change in preferred strategies during competition. Skating evolved over time from traditional Classic skiing by adding a one-sided outward leg push, and it is now prohibited during Classic races. In recent years, elite skiers have developed their upper body strength to such an extent that, in Classic races, they can use Skating skis exclusively with the double-poling (DP) technique, particularly for long distances (20). In order to generate more upper body power, skiers have shown increases in upper body range of motion and wind up (shoulder flexion and trunk extension) to generate more force during pole-plant (1).

In addition to the technique results, a number of additional kinematic factors were identified as having high variance across skill level. Specifically, the frontal area of elite skiers is smaller, resulting in less aerodynamic drag force. This indicates that higher skill level skiers were able to maintain a smaller frontal area and are able to ski at faster speeds with greater efficiency. Previous studies have found a relationship between decreased drag from lower air resistance being related to lower demand on propulsive force, lower metabolic cost and lower oxygen cost to maintain the same speeds (21). Further, elite skiers had a decreased external rotation angle of the skis while gliding compared to the beginners who skied with their skis more externally flared. A decreased external rotation of the ski angle could suggest lower braking forces throughout the trial resulting in greater speed due to reduced friction from the ski-snow interaction (22). Notably, the SVM classifier trained within the current study complements those presented previously using head-mounted GNSS data (23) and obtained comparable performance. This suggests that although similar cycle classification can be obtained with fewer sensors, the current approach has the added benefit of full body 3D animation to administer actionable feedback to improve on the execution of each sub-technique.

Although this study is novel in many ways, due to the field-based nature of the results reported, there are several limitations to consider. One notable limitation of this study is that only eight participants were recruited for the analysis, all of whom had varying skill levels and came from different demographic groups. This includes the varied distributions of sexes and ages across different skill groups. This small sample size may limit the generalizability of the results to a broader population of skiers. Further, this study only focuses on the biomechanical factors associated with cross-country skiing performance and may overlook factors beyond mechanical changes to technique that may have an impact on performance outcomes. Participants were not instructed to use a specific technique for navigating the track and could decide which techniques to use on their own. This may have affected the comparison of movements between the athletes as all of the segments were compiled together despite variations in techniques used. In addition, out of the 5,568 cycles generated, only 241 were manually labelled to train the SVM classifier. Although this an undoubtedly more efficient means of evaluating propulsion strategies across a large breadth of data, this may have created bias for certain techniques despite the AUC statistic reaching 0.94, suggesting strong fit statistics. Certain external factors that may have affected the gliding performance of the skis were not accounted for, such as ski specifications and snow conditions, further, turning mechanics were not considered for the current study. Finally, it is worth noting the potential effects of both artificial stimulus, and wearable sensor drift. Although the wearable sensors used here facilitated the acquisition of large quantities of over-ground skiing movement, their body-worn nature may have affected skier movement. Further, although care was taken to correct for sensor drift (Section 2.5) through careful debiasing, segmentation, and re-alignment, the results here may be affected by some degree of drift.

This study provides valuable insights on the biomechanical differences between skill levels of cross-country skiing athletes and contributes to the body of literature on full-body cross-country kinematics during natural over-ground skiing movements. Data reduction techniques such as PCA may be used to identify factors significantly related to skiing performance and can be used in sport-field settings. This framework used in this analysis to evaluate human movement can be applied across a variety of sports to identify factors relating to performance and determine differences between skill levels. This model can also be applied using data captured from a marker-less video-driven setup which allows for it to be used by coaches and athletes outside of a research setting. Coaches can use the current model as a framework for evaluating and comparing developing athletes to examine how their technique compares to a higher skill athlete. From this analysis, it can be determined that selection of technique, decreasing drag by maintaining a small frontal area and reducing ski external rotation, and increasing upper and lower body power production were all factors that varied with skill level.

## Data Availability

The raw data supporting the conclusions of this article will be made available by the authors, without undue reservation.
